# Supplementary material for: A Qualitative Study of Health-Related Experiences Associated with Lifestyle Role Transitions Among Local Residents in Their 60s
Source: Healthcare (Basel). 2025 Oct 26;13(21):2702. doi: 10.3390/healthcare13212702 (PMC12607828; doi:10.3390/healthcare13212702)
Supplement: Supplementary file 1 [file healthcare-13-02702-s001.zip › healthcare-3852409-supplementary.pdf]

Supplementary Material S1: Interview guides

Thank you for participating in today's interview.

The purpose of this interview is to hear about roles and health-related activities within each group: employment, grandchild care, and caregiving. Each group will consist of 4 to 6 people. The interview will last 60 to 90 minutes. We will ask for your opinions on the given themes. To accurately record everyone's comments, we will record the interview using an IC recorder if you consent. We will follow an interview guide, but you may decline to answer any questions you find difficult. Please promise not to discuss anything heard in today's interview elsewhere.

Interview questions

【Daily Life and Life Events】

1. Have there been any major life events since you entered your 60s?
2. Did these major life events lead to changes in your daily patterns or roles?
3. Please tell me why you are currently working, caring for grandchildren, or providing caregiving. What meaning do these activities hold for your life?

【Physical Condition】

1. Do you currently experience any noticeable symptoms?
2. Are there times when the symptoms mentioned in 1 feel particularly strong?
3. Are you actively engaged in any health-promoting activities?
4. If so, please tell me why you are doing them.

【Perceived Fatigue】

1. Regarding the fatigue you currently feel, when do you feel like the fatigue doesn't go away?
2. If there are things you consciously do to recover from fatigue, please tell me.
3. What would you like to pay attention to in order to live a healthier life going forward?

【Social and Environmental Situation】

1. Do you participate in any hobbies, volunteer work, etc.? What meaning do these activities hold for your life?
2. Please share any thoughts you have about your living situation.
